# ENGAGING DIVERSE OLDER ADULTS IN AGING RESEARCH: IDENTIFYING COMMUNICATION AND COMMUNITY ENGAGEMENT STRATEGIES

**DOI:** 10.1093/geroni/igad104.3302

**Published:** 2023-12-21

**Authors:** Trudy Gaillard, Dawn Carr, Julia Sheffler, Ursula Weiss, Donna Neff, Neil Charness, Fern Webb

**Affiliations:** Florida International University, Miami, Florida, United States; Florida State University, Tallahassee, Florida, United States; Florida State University College of Medicine, Tallahassee, Florida, United States; Florida State University, Tallahassee, Florida, United States; University of Central Florida, Orlando, Florida, United States; Florida State University, Tallahassee, Florida, United States; University of Florida, Gainesville, Florida, United States

## Abstract

A growing challenge is ensuring that diverse older adults participate in research studies. If studies are not inclusive of diverse populations, it is not possible to determine if interventions are likely to be universally beneficial or reduce health disparities. Smaller and more regional studies are particularly likely to struggle to recruit and retain diverse older adults for their research. This project uses quantitative and qualitative data based on a new collaboration consisting of independent scholars at four research institutions associated with three participant research registries on aging in Florida. The aims of this project are to: 1) describe characteristics of aging-focused research registrants, and 2) share communication strategies for recruitment specifically designed to increase participation of a variety of underrepresented populations, including minority, rural, and under-served. Preliminary results show that it is important to specifically adopt culturally informed recruitment strategies given its direct result in adequate representation from diverse and under-represented groups. All three participating registries are currently enrolling persons and seek a specific diverse demographic which captures the racial, cultural and geographic variation observed in residents throughout Florida. For example, these registries seek individuals who self-identify as African American/Black, Caribbean, Hispanic/Latino adults aged > 55 years; persons living in rural communities, and persons who have survived cancer or are interested in cancer-related research. We describe current infrastructure employed at each institution as well as recommendations in building long-term trust across each collaborating institution. Finally, we share an innovative model leveraging strengths and opportunities for statewide engagement across each registry.

